# Residents: admissions, training and assessment

**Published:** 2015-12-11

**Authors:** Kalyani Premkumar

**Affiliations:** University of Saskatchewan, Saskatoon, Saskatchewan

Undoubtedly, residents play a very important role in the healthcare system of most countries. The process of admission of medical trainees into specialties, residency training and their assessment are key determinants of the future of healthcare.

In Canada, according to the 2014–15 data, there were 13,439 residents (excluding Fellows) distributed in 42 specialties/sub-specialities.^1^ Every year, there are 3,280 positions in Canada. About 10% of these residents are International Medical Graduates (IMGs) or Canadians studying abroad (CSAs). Residents are subjects of various investigations exploring areas such as sleep deprivation, stress,^2^ medication errors to differences between Canadian Medical graduates (CMGs), IMGs and CSAs that can help with decisions of policy makers.^3^ Here I dwell on a two interesting examples of studies using residents as subjects.

Philips and Barker who systematically examined the occurrence of fatal medication errors over 25 years in the US attribute a significant spike in medication errors in July to the entry of a new cohort of medical residents.^3^ Can changes in medical education have an impact on such medication errors? While this needs to be investigated, better orientation of final year medical students for entry into residency, closer supervision of new residents, and training of supervisors on providing feedback to residents are some measures that ought to be seriously considered. The introduction of Competency by Design (CBD) by the Royal College of Physicians and Surgeons is definitely an important step in this direction.^4^

A recent study by Curtis and Dube looked at the characteristics of CMGs, IMGs and CSAs in an era where measures are being taken to alleviate physician shortage.^5^ IMGs make up about 25% of practicing physicians in Canada. However, the scenario is changing, with more CSAs applying for residency positions. As of 2014, there were 3600 CSAs. These findings in particular have implications for policy: IMGs are less likely than CMGs to report that they intend to stay and practice in Canada; CSAs are less likely to report their willingness to practice in the region where they completed their residency. CSAs and IMGs are more likely to choose family medicine than CMGs. Given the paucity of residency positions in Royal College specialties, and less inclination of CMGs to choose family medicine, do changes have to be made to admit more CSAs and IMGs to fill family medicine positions? If that is done, how can IMGs be enticed to practice in Canadian regions where physicians are most needed? IMGs and CSAs are less likely to be females. How will this impact the diversity and equality that Canada tries to promote? These are interesting and complex questions that arise from the study.

## This publication of CMEJ focuses on residents

In Canada, the Canadian Resident Matching Services (CARMs), a non-profit, fee-for-service organization, facilitates a fair and transparent process of resident admissions into programs.^6^ Medical trainees begin the online application process by uploading transcripts and supporting documents along with their ranking of choices of specialties and programs. Individual programs across the country access these documents and based on their criteria, call residents for interviews and upload their ranking of students. CARMs matches the student choices with specialties and programs using an algorithm. The interview process by programs is a vital element to the CARMs process. Sklar et al. explore the differences in scores of applicants to postgraduate admissions using traditional interviews (TIs) and Multiple Mini Interviews (MMIs). While the scores correlate well, the rank order varies. The authors suggest that MMIs and TIs measure different (so called) non-congitive areas.*

The Royal College of Physicians and Surgeons and the College of Family Physicians of Canada play are regulatory bodies that oversee the quality of residency training in Canada. Research into the training process and application of educational principles helps promote training quality. In this edition, Guajardo and colleagues examine the effect of inclusion of patient name and image in virtual patient cases on knowledge acquisition.

A large part of resident training involves role modeling by preceptors. In Canada, with its wide distribution of population in rural and remote areas, medical schools train students and residents in these areas under the supervision of preceptors. Piggott, Morris, and Lee-Poy examine the facilitators and barriers to engagement of preceptors in distributed medical campuses. There are several barriers that can be addressed by medical schools such as: provision of training of preceptors in being better teachers, ensuring that preceptors in these areas are aware of curricular objectives, among others. Lubitz, Lee, and Hillier look at the perception of residents to the longitudinal integrated curriculum, a form of training provided in mostly rural areas.

With the introduction of the CanMEDs roles, residents are trained and assessed using the CanMEDs framework.^7^ In-training Evaluation Reports (ITERS) is an accepted assessment mechanism that is utilized by most Canadian medical schools. ITERs^8^ require preceptors to provide feedback to residents and the program using a Likert scale and comments under the categories of Residents as Medical Experts, Communicators, Collaborators, Leaders, Health Advocates, Scholars and Professionals. Patel et al explore the perception of pediatric residents and faculty of using ITERS. There is considerable debate on what is/are the best tool(s) to evaluate each of the CanMED roles. Poulton and Rose review the importance of Health Advocacy in resident training, current attitudes, and issues relating to its evaluation.

A large volume of literature exists in relation to the transformation of trainees from novices to experts.^9^,^10^,^11^ Illness scripts are a recognized mechanism that help physicians integrate new incoming information with existing knowledge, recognize patterns and irregularities in symptom complexes, identify similarities and differences between disease states, and make predictions about how diseases are likely to unfold.^11^ Expert physicians have a wealth of formulated illness scripts that help them make quick and accurate diagnosis. Resident curricula are planned and designed to provide trainees opportunities in various forms to promote formation of illness scripts. In this issue that focuses on residents, Lubarsky and colleagues expand on the script theory to cultivate illness script formation and Phang and colleagues explore the mental mechanisms used by residents to estimate disease probability.

## Footnotes

* We at the CMEJ are dissatisfied with the common use of the terms cognitive and non-cognitive. Please see the editor’s note in the Sklar et al article and read the editorialette in this issue.
